# What can clinical disorders involving the basal ganglia tell us about the pathophysiology of obsessive and compulsive symptoms?

**DOI:** 10.1007/s00702-026-03226-9

**Published:** 2026-08-04

**Authors:** Violette Chereau, Benjamin R. Underwood

**Affiliations:** 1 School of Clinical Medicine, University of Cambridge, Box 111 Cambridge Biomedical Campus, Cambridge, CB2 0SP UK; 2 Department of Psychiatry, University of Cambridge, Herchel Smith Building, Forvie Site, Robinson Way, Cambridge, CB2 0SZ UK; 3 Cambridgeshire and Peterborough NHS Foundation Trust, Windsor Unit, Fulbourn Hospital, Cambridge, CB21 5EF UK

**Keywords:** Obsessive-compulsive symptoms, Basal ganglia disorders, Parkinson’s disease, Tourette’s syndrome, Huntington’s disease

## Abstract

The neurobiology and pathological mechanisms underlying obsessive-compulsive symptoms have been explored in the literature for more than fifty years without reaching a definitive conclusion. What is clear is that some brain areas, including the basal ganglia and connected structures, have been implicated through several different approaches, including use of animal models and examination of human subjects with different cerebral lesions and pathologies. The purpose of this narrative review is to explore how examination of disorders affecting the basal ganglia can give insights into the pathophysiology of obsessive-compulsive symptoms and obsessive-compulsive disorder. These clinical conditions include Parkinson’s disease, Huntington’s disease, paediatric autoimmune neuropsychiatric disorders associated with streptococcal infections (PANDAS), Sydenham’s chorea, and Tourette’s syndrome. We review neuroimaging, genetic, and clinical studies in these conditions. Overall, the literature discussed supports the idea that the reward circuitry, including the limbic and lateral orbitofrontal loops of the basal ganglia, as well as goal-directed versus habitual pathways in the dorsal striatum, are involved in obsessive-compulsive disorder and obsessive-compulsive symptoms.

## Introduction

### A brief introduction to obsessive–compulsive disorder

Obsessive–compulsive disorder (OCD) can be a deeply debilitating condition, ranking 10th in WHO’s 10 leading causes of disability (Bobes et al. 2001). It is also common, affecting an estimated 2% of the worldwide population (Sasson et al. 1997). The disorder is characterised by obsessions – unwanted and persistent thoughts, images, and urges – and/or compulsions – repetitive behaviours aimed at reducing stress (Pigott 1998). The seriousness of the problem underlines why understanding the underlying mechanisms of OCD is vital to improving the quality of life of those living with the condition, and might allow us to develop novel, more targeted and effective treatment options.

Most individuals experience passing, fleeting intrusive thoughts, but importantly in OCD these cause distress and/or impairment of function. OCD has historically been viewed as a cyclic condition, whereby obsessive thoughts generate anxiety which is alleviated in the short-term by compulsions – though more recent theories suggest that obsessions may instead be a *post hoc* explanation for compulsive behaviour (Gillan et al. 2014).

Despite stereotypes of individuals with OCD engaging in contamination fears and compulsive washing or cleaning behaviour, the condition can in reality widely vary in its expression, for example including obsessions revolving around taboo or ‘forbidden thoughts’ and subsequent reassurance-seeking behaviour (Aouizerate et al. 2004).

The basal ganglia have long been implicated in the origin of obsessive and compulsive symptoms, as the basal ganglia are involved in such processes as evaluating and learning about goals and risks, and directing behaviour (Lanciego et al. 2012). This is reflected in some psychological models for OCD, notably the learning and cognitive behavioural views (Rachman and de Silva 2009). The former is based on Mowrer’s two-factor theory of avoidance and fear (Mowrer 1939). It attributes OCD to a maladaptive learning process, whereby an individual learns through classical conditioning that certain stimuli cause distress, while learning in conjunction through operant conditioning that particular behaviours alleviate said distress. The individual therefore engages in behaviours (compulsions) directed towards reducing an aversive internal state (the obsession). In most cases this is an adaptive response, however, in OCD the response becomes habitual and over-performed even though the form the behaviour takes may be entirely unrelated to the current source of stress, especially if the fear driving the response is irrational.

The cognitive behavioural view of OCD builds upon the learning view, by adding a cognitive component and accounting for the role of the patient’s beliefs and thoughts in the development of the condition. We can see therefore how both of these models might implicate dysfunction in the basal ganglia, given the roles outlined above (Lanciego et al. 2012).

A further possible psychological explanation for the development of these symptoms is that OCD is a form of behavioural and cognitive addiction, whereby compulsions become addictive due to their ability to transiently reduce the aversive internal state which is anxiety (Figee et al. 2011). Given its role in drug and other forms of addiction, as well as its position as the reward and reinforcement circuitry of the brain, this view would also be consistent with basal ganglia dysfunction in the basis of OCD.

### A brief introduction to the basal ganglia

**Fig. 1:**
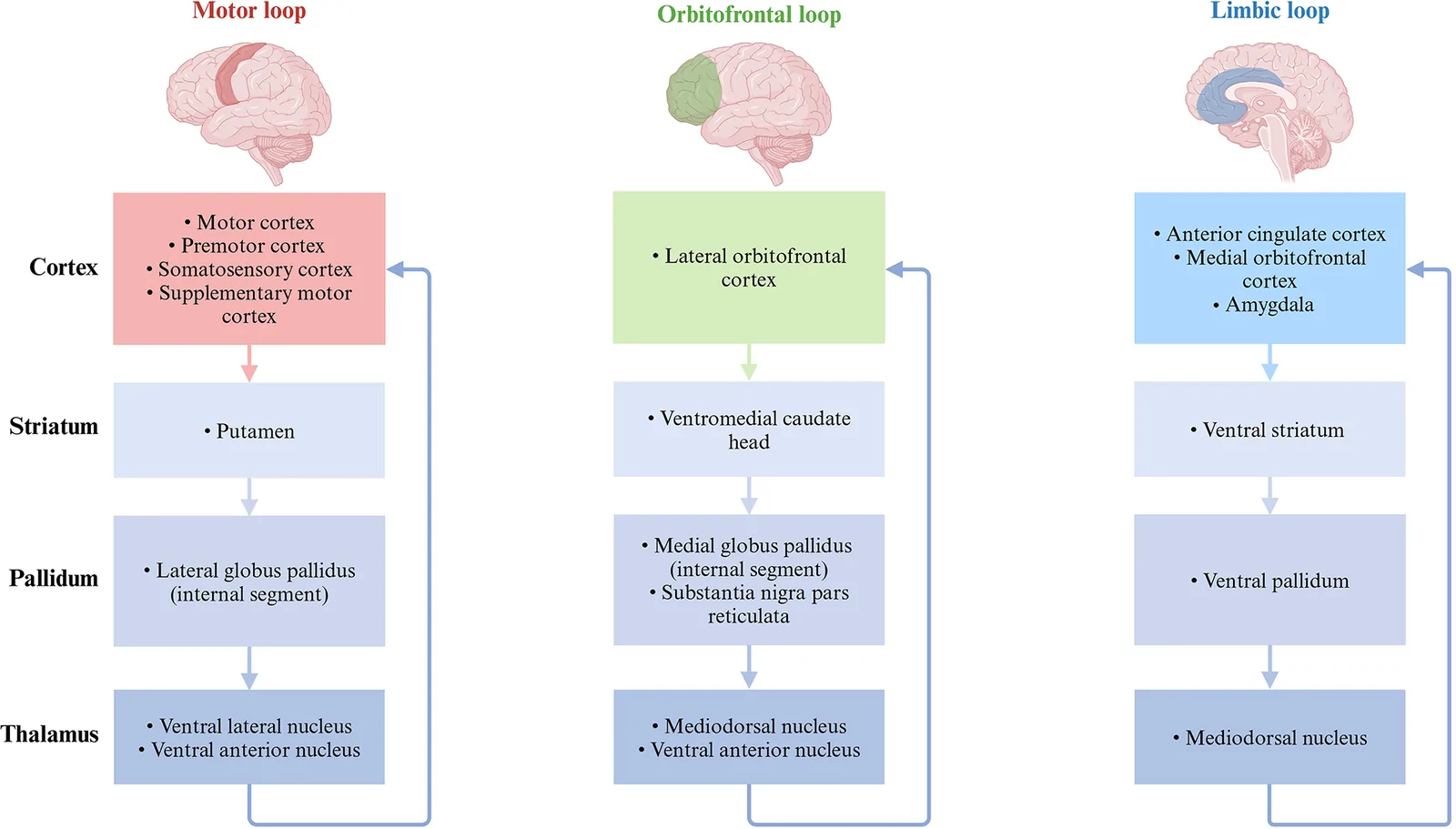
A schematic of the motor (red), lateral orbitofrontal (green), and limbic (blue) loops of the basal ganglia. Arrows indicate the flow of information from one brain region to the next. Adapted from ‘Basal Ganglia Motor and Non-motor Loops’, by BioRender.com (2024). Retrieved from https://app.biorender.com/biorender-templates. Adapted from https://app.biorender.com/biorender-templates

The basal ganglia are a set of subcortical structures including the striatum (made up of the caudate, putamen, and nucleus accumbens (NAc), globus pallidus (GPi/e), subthalamic nucleus (STN), substantia nigra (pars compacta and reticulata, SNc and SNr), and ventral tegmental area (VTA) (Macpherson and Hikida 2019). These are coordinated in a series of five parallel cortico-basal ganglia-thalamo-cortical (CBGTC) loops which can be broadly divided into three groups: motor (consisting of the motor and oculomotor circuits), associative (lateral prefrontal (PFC) and lateral orbitofrontal (OFC) loops), and limbic (one loop relating to the anterior cingulate (ACC), ventromedial prefrontal cortex, and medial orbitofrontal cortex, as well as other subcortical structures) (Fig. 1) (Alexander et al. 1986).

**Fig. 2:**
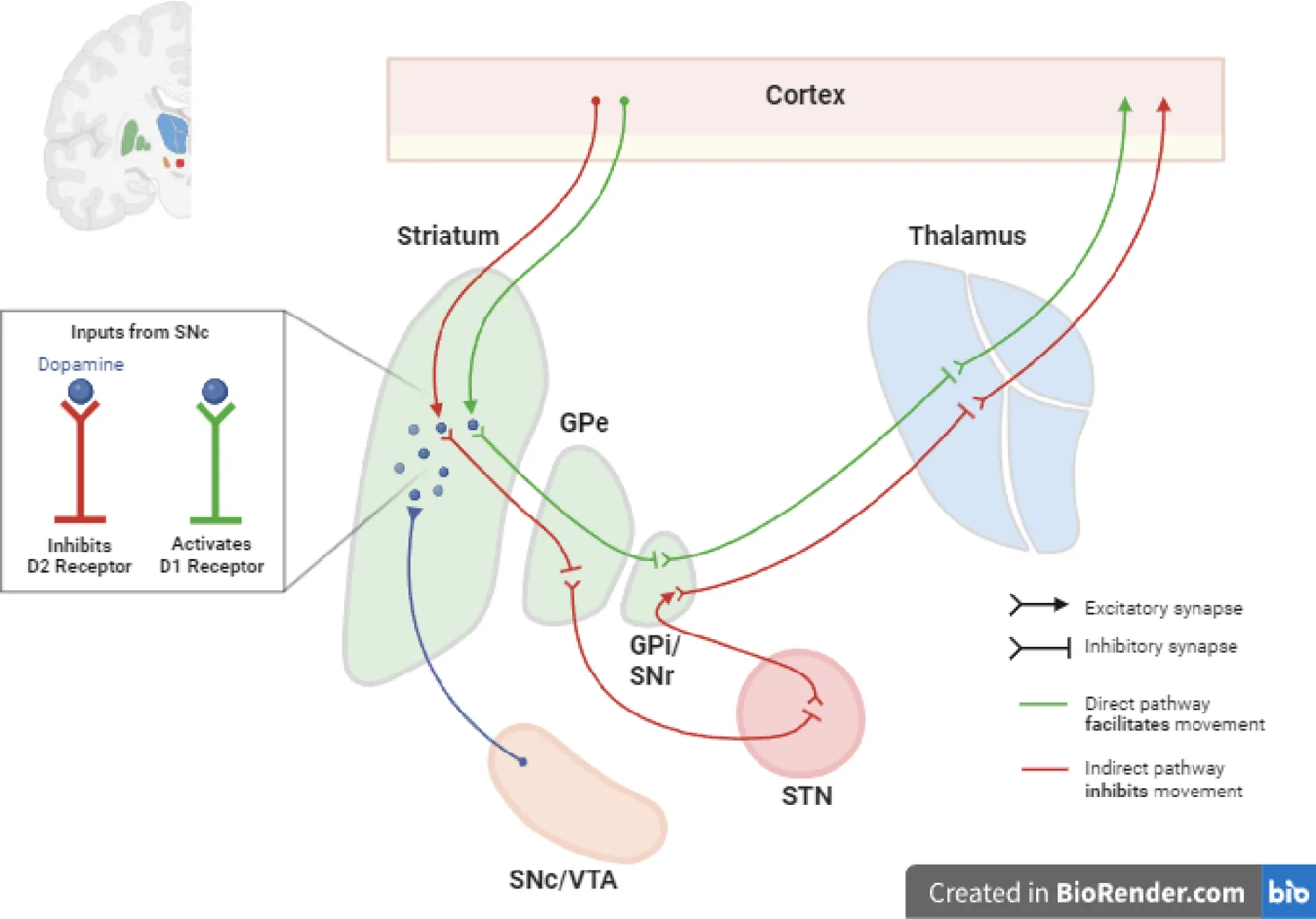
A diagram of the direct and indirect pathways of the basal ganglia. Lines with arrowheads indicate an excitatory connection while lines with flat heads indicate an inhibitory one. The green and red lines depict direct and indirect pathway connections respectively. There is also a smaller diagram showing, in greater detail, the effects of dopaminergic inputs from the SNc on each of the basal ganglia pathways. Adapted from ‘Direct and Indirect Pathways of the Basal Ganglia’, by BioRender.com (2024). Retrieved from https://app.biorender.com/biorender-templates. Adapted from https://app.biorender.com/biorender-templates

In all of these circuits, the striatum is the main input, receiving glutamatergic fibres from the cortex (Lanciego et al. 2012). From the striatum, the information can pass through either a direct or an indirect pathway (DeLong 1990) (Fig. 2). In the former, GABAergic striatal neurons project monosynaptically to the GPi/SNr, while in the latter GABAergic striatal neurons project to the GPe first, which in turn forms inhibitory projections to the STN. The STN then sends excitatory fibres to the GPi/SNr. It is therefore at the GPi/SNr that the two pathways converge, and GABAergic fibres project to the thalamus which itself has excitatory projections towards the cortex (Gerfen and Bolam 2010). The overall effect of these circuits is that the direct pathway excites an area of cortex (determined by the particular parallel loop in question), while the indirect pathway inhibits it (Nambu 2017). There is also evidence for a hyperdirect pathway (Nambu et al. 2002), however this lies outside the scope of this review.

The striatal projections contributing to the direct and indirect pathways are under modulation by neurons from the SNc, which release dopamine (DA) (Bolam et al. 2000). This DA binds either to excitatory D1 receptors in the direct circuit or inhibitory D2 receptors in the indirect circuit, its release therefore having an overall excitatory effect on the cortex (Gerfen and Surmeier 2011). In contrast, the VTA provides the principal modulatory dopaminergic innervation to the ventral striatum (VS) (Dafny and Rosenfeld 2016), and therefore particularly plays a role in the limbic loop of the basal ganglia. Despite being otherwise largely similar in structure to the other loops, its direct and indirect pathways are less demarcated (Alexander and Crutcher 1990). As such, all loops start with excitatory input from the cortex to the striatum (in this case the VS, including the NAc and medial part of the caudate), but in the limbic loop the VS then makes inhibitory connections to the ventral pallidum, which in turn projects to the thalamus (Haber 2011).

Historically, the basal ganglia have been primarily associated with motor control and action selection, however, with increased understanding of the functionally distinct parallel loop circuitry, its known functions have expanded to include motor (and other forms of) learning, executive function and behaviour, motivation, and emotion (Lanciego et al. 2012). The functions can also be divided in terms of striatal anatomy, looking at the dorsolateral (DLS, corresponding with the putamen), dorsomedial (DMS, caudate), and ventral (medial caudate, olfactory tubercle, and nucleus accumbens) aspects. The DLS receives afferent projections from sensorimotor cortices, and therefore is heavily implicated in the functioning of the motor loops as well as habit formation. The DMS meanwhile gets its input primarily from frontal- and parietal-associated cortices, hence its principal role in the associative loops and it being linked to goal-directed instrumental learning (Balleine et al. 2007). Finally, the VS is innervated by limbic structures including the ACC, amygdala, hippocampus, and medial prefrontal cortex, equipping it for a role in emotional processing and learning about stimuli predictive of rewards and punishments. (Macpherson and Hikida 2019).

Despite the functional and anatomical segregation between the parallel loops of the basal ganglia, motor, cognitive and emotional responses need to be linked in some way to act in concert (as they indeed do, especially in motivated behaviour). This link may be subserved by an ascending spiral of striatonigrostriatal pathways connecting the limbic loop with the associative and motor ones, such that emotions can modulate cognition and action (Haber et al. 2000).

One current theoretical model involves thinking of the DS and VS as an actor and a critic respectively, whereby the former selects which actions to perform to achieve a goal based on particular internal and external stimuli, as well as its store of innate species-specific motor programmes. The VS meanwhile provides immediate feedback, assigning value to certain actions and indicating whether this strategy should be re-adopted in similar future conditions (Joel et al. 2002). The critic is thought to work by computing prediction errors, largely through dopaminergic signalling. Positive prediction errors are met with phasic bursts of dopamine firing from the VTA, and negative prediction errors are signalled by pauses in DA firing.

Given the numerous different functions subserved by the basal ganglia, it follows that disorders of these structures might be widely heterogeneous in their expression, especially depending on which particular pathway or area is affected. This review focuses on Parkinson’s (PD), Huntington’s (HD), Sydenham’s chorea (SC), paediatric autoimmune neuropsychiatric disorders associated with streptococcal infections (PANDAS), and Tourette’s syndrome (TS), each of which is thought to be a result of dysfunction in distinct structures within the basal ganglia.

### The aims and structure of this review

Here we review obsessive and compulsive symptoms observed in OCD compared to other basal ganglia disorders to understand what role basal ganglia dysfunction might have in OCD and how it comes about. We chose a narrative format for this paper given the exploratory nature of our research question and lack of research surrounding it, which demands a level of detailed and nuanced interpretation that we felt was best achieved with a narrative rather than systematic approach. We start by exploring the evidence for the roles of the basal ganglia in idiopathic OCD, followed by similarities in neuroimaging and treatment with other disorders associated with obsessive-compulsive traits. In summarizing the findings of this review, it will attempt to answer three main questions:

1. What does this evidence tell us about the role of the basal ganglia in OCD?
2. Can we separate the neurological mechanisms behind individual obsessive and compulsive symptoms?
3. What implications may this have for future OCD treatments?

## Literature review

### Evidence

#### Evidence for a role of the basal ganglia in idiopathic OCD

Outside of the evidence for obsessive and compulsive comorbidity in various basal ganglia disorders, it is important to consider data implicating these structures in idiopathic OCD. The evidence discussed here falls under two categories: neuroimaging studies, and surgery or other treatments. There are also animal models for the condition, and brain lesion studies which largely align with the findings detailed below, however, these lie outside the scope of this review (for examples see Burguière et al. 2013; Ahmari et al. 2013; Laplane et al. 1989).

There is now a substantial amount of neuroimaging data indicating that dysfunctional reward circuitry could play a role in developing OCD, particularly the orbitofrontal and limbic loops (Figee et al. 2016). One meta-analysis of MRI studies found that, compared to healthy controls, OCD patients had a smaller left ACC and smaller OFC bilaterally (Rotge et al. 2009), while a separate MRI study comparing 26 OCD patients to 26 healthy controls found significantly smaller caudate volumes (Robinson et al. 1995). A PET imaging study found increased metabolic activity bilaterally in the OFC compared to healthy controls (but not depressed controls), as well as in both caudate nuclei (Baxter et al. 1987). It is important to acknowledge where studies contrast in their findings, however, with reports on children with OCD generally indicating larger ACC volumes than controls (Rosenberg and Keshavan 1998) while differences in ACC volume for diagnosed adults vary widely across studies. Nevertheless, the ACC has consistently been found to be hyperactive in OCD, therefore the issue may lie more in activity than in structure (Maia et al. 2008).

Other studies have explored the activity of these highlighted areas in tasks that would traditionally involve the basal ganglia rather than simply at rest: for example, when engaging in a monetary reward task, it was found that patients with OCD show reduced reward anticipation, and decreased activation of limbic loop circuitry and the NAc compared to controls (Figee et al. 2011). Additionally, in a meta-analysis of the results of OCD symptom provocation paradigms, almost all studies showed activation of the ACC and OFC loops, further reinforcing the roles of these circuits in the condition (Rotge et al. 2008). Changing focus to other striatal areas, Isobe et al. (2021) found a correlation between impaired cognitive flexibility and abnormal putamen morphology in an analysis of MRI data from both OCD patients and their asymptomatic relatives. Others have looked at dopaminergic systems, for example, Denys et al. (2004) in their review suggest increased DA concentrations in the striatum relative to healthy controls.

Successful treatment options can also provide a helpful framework with which to approach the problem of determining possible pathophysiology for OCD: if targeting a particular aspect of the brain circuitry improves symptoms, it follows that that area was at least in part responsible for the dysfunction, or related to it (although it is also possible that the treatment provides compensation for a faulty network rather than reflecting the cause of the issue).

Some of the earliest treatments for OCD, still in use today as first-line care, are talking therapies, particularly a form of cognitive behavioural therapy called exposure-response prevention (ERP). It is based on a framework of sessions in which patients are confronted with their fears (obsessions) and refrain from engaging in compulsions. Using Mowrer’s two-factor theory of fear and avoidance (introduced in Sect.  “A brief introduction to obsessive-compulsive disorder”) applied to classical theories of OCD (Mowrer 1939), we can see how this form of therapy may be useful for patients: it is a way of achieving extinction, attempting to break the cycle of obsession and compulsion by preventing the latter. Meanwhile, the therapist can work with the patient to target the cognitive components of the disorder and challenge the fears driving the obsessions (Hezel and Simpson 2019).

Neuropharmacology is another treatment modality, often used in conjunction with OCD to further improve outcomes. Drugs such as clomipramine and fluoxetine that block serotonin reuptake, thus increasing serotonin signalling in the brain, are typically used, leading to a serotonergic hypothesis of OCD (Insel et al. 1985). Another neurotransmitter that is heavily implicated in the basal ganglia’s functions is DA, and testing the effects of dopaminergic drugs in OCD has been examined. Murray et al. (2019) studied OCD patients engaging in reward-processing tasks while being administered either a DA agonist or antagonist. Unexpectedly, they found that both drugs reduced abnormal increases in ACC activity seen in OCD patients experiencing negative prediction errors – so while this necessitates more research, it does indicate some role for abnormal DA signalling in OCD and some potential for dopaminergic treatments in future.

More recently, deep brain stimulation trials have proven to be successful for treatment-refractory OCD, particularly ones involving the NAc (Denys et al. 2010) – which is in line with the finding that the NAc is less active in OCD patients during reward-based tasks. A meta-analysis of deep brain stimulation studies further supported strategies targeting the NAc, as well as the anterior limb of the internal capsule (between the caudate and lentiform nucleus), inferior thalamic peduncle, and subthalamic nucleus (Kohl et al. 2014).

In certain extremely resistant cases where other forms of treatment have not worked, a neurosurgical approach may be taken (Nuttin et al. 2014). Most often, this will involve cingulotomy (removal of the ACC), but there has also been success historically with ablative surgeries to the anterior limb of the internal capsule, and subcaudal areas (Balachander et al. 2019). Regardless, all of these options are likely to disrupt frontrostriatal connections which may underlie OCD.

#### Evidence for obsessive and compulsive symptoms in Parkinson’s disease

**Fig. 3:**
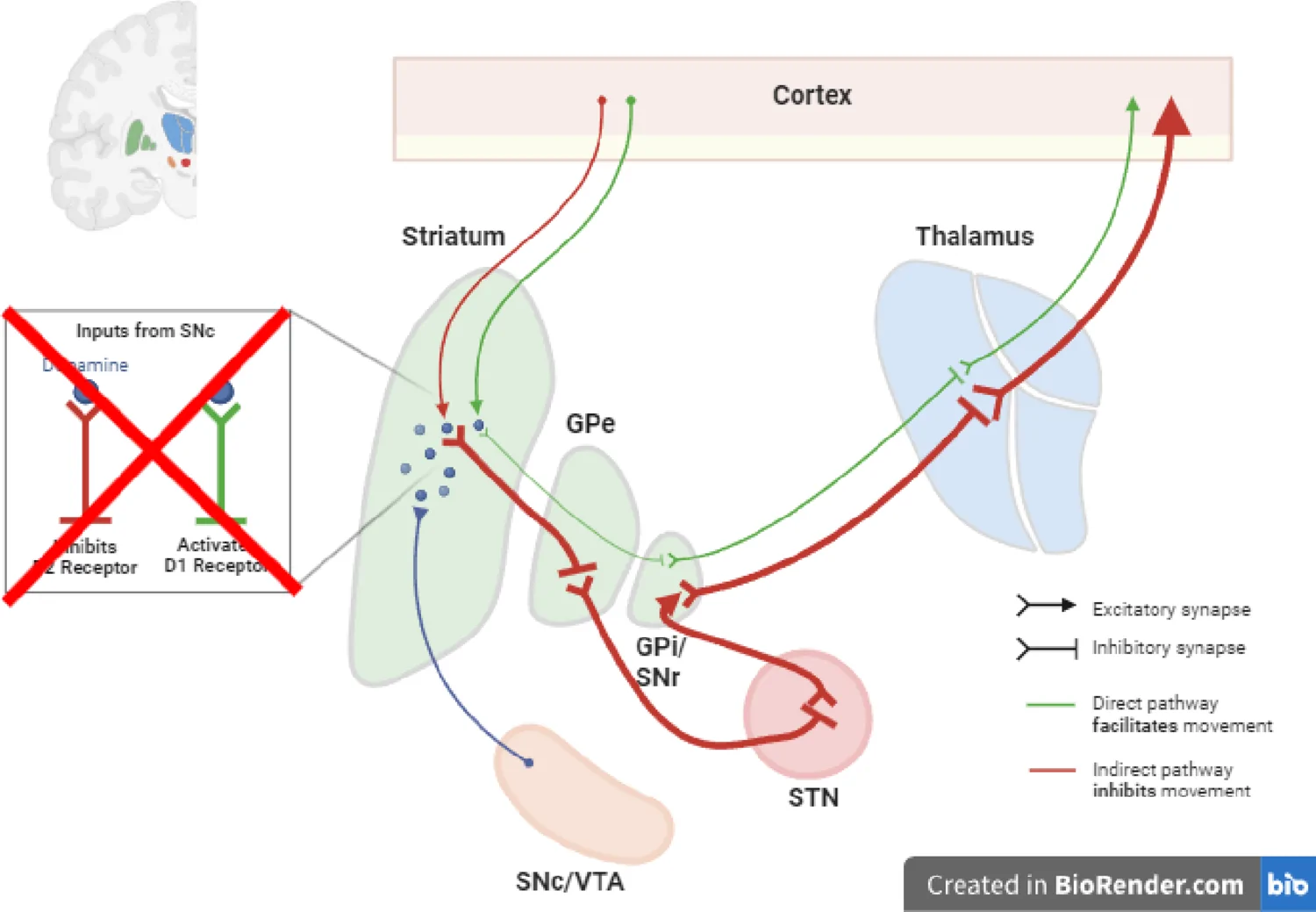
A schematic showing the pathological changes to the basal ganglia in Parkinson’s disease. Thicker lines indicate greater activity, while thinner lines indicate attenuated activity. The cross over the SNc input diagram indicates a lack of communication from this brain region. Adapted from ‘Direct and Indirect Pathways of the Basal Ganglia’, by BioRender.com (2024). Retrieved from https://app.biorender.com/biorender-templates. Adapted from https://app.biorender.com/biorender-templates

PD is one of the most common disorders of the basal ganglia. It is characterised by a triad of cardinal symptoms: tremor at rest, rigidity, and bradykinesia (Parkinson 1817). Several non-motor symptoms have also been identified, most commonly dementia and depression – and in some cases, there are symptoms reminiscent of OCD. The condition arises as a result of progressive degeneration of the dopaminergic neurons of the substantia nigra pars compacta, leading to reduced inhibition of the indirect pathway of the motor loop and reduced excitation of the direct pathway (Fig. 3). Thus, there is overall inhibition of the thalamus and decreased activation of the motor areas of the cortex, which leads to motor difficulties, particularly in action selection and initiating voluntary movements. Psychiatric symptoms in PD meanwhile may result from this general dysfunction of basal ganglia loops secondary to neurodegeneration, or indeed they may be iatrogenic secondary to treatment (Marsh 2000).

Given the overlap in affected regions in both OCD and PD, a number of studies have attempted to identify links between the two. However, findings have been mixed. One group found that obsessive-compulsive personality disorder is the most common personality disorder in PD patients (Nicoletti et al. 2013), while Tomer et al. (1993) showed that the severity of left-handed motor symptoms (indicating right basal ganglia dysfunction) in PD patients was a reliable predictor of certain obsessive-compulsive traits including repetition, disturbing thoughts, and obsessions of cleanliness (further corroborated by Maia et al. 2003). This is in line with another study (Mataix-Cols et al. 2004) that found that patients with contamination or cleaning OCD displayed greater activation of the right caudate relative to controls (Kummer and Teixeira 2009), indicating perhaps a hemispheric bias in the development of OCD (or at least the contamination subtype). There is also some evidence for a temporal gradient of obsessive-compulsive symptoms (OCS), with patients with more severe PD (and therefore an older diagnosis) presenting with significantly more traits compared to controls, in contrast with milder PD patients, who did not (Alegret et al. 2001).

Post-encephalitic Parkinsonism in particular has been associated with the development of obsessive-compulsive characteristics. This may be because it tends to be earlier in onset (Dhawan and Chaudhuri 2007), hence providing a longer time for individuals to develop OCD-like symptoms (in accordance with the Alegret et al. (2001) proposition). Another interpretation may be that, as it is a result of viral infection which affects a more widespread area, other structures outside of the substantia nigra may be affected that cause OCD-like symptoms (Maia et al. 2003).

In contrast, other studies showed no relationship between the severity of PD and obsessive-compulsive traits (Müller et al. 1997; Harbishettar et al. 2005). This indicates perhaps the involvement of different circuit abnormalities within the basal ganglia in the pathology of OCD as compared to PD.

Consideration has also been given to the effects of treatment on obsessive-compulsive traits in PD. Certain repetitive behaviours in PD have been noted to likely be of iatrogenic origin – notably punding, which consists of repetitive stereotyped and seemingly purposeless complex behaviours (Rajalingam and Fasano 2023). Prior to the introduction of dopaminergic therapy for PD, discussions of obsessive and compulsive traits in the disorder were far less common, suggesting these therapies may be playing a part in their appearance. This may imply a role for excessive dopaminergic signalling in OCD (particularly compulsions, as obsessions seem to be absent in punding), or indeed they may be phenomenologically distinct (Kummer and Teixeira 2009; Voon 2004). This former idea is also reviewed by Jankovic (2008), who posits that hedonistic homeostatic dysregulation (including symptoms such as craving, binge eating, and other compulsive behaviours) is associated with dopamine dysregulation syndrome due to DA agonist use. This would support claims for a ‘behavioural addiction’ view of compulsions, given the role of DA in addiction (Voon et al. 2017).

Data from deep brain stimulation studies, where stimulation of the STN can improve symptoms of both OCD and PD, may shed further light on the relationship between PD and OC symptoms (Lee et al. 2019). Mallet et al. (2002) conducted a study implanting subthalamic electrodes to alleviate Parkinson’s symptoms in two patients who also had a history of severe OCD before the development of PD. They found that this improved disability from both conditions. However, it is important to acknowledge that pathways do overlap in the subthalamic nucleus for both the motor and associative/limbic loops, so results may be due to this phenomenon rather than any greater overlap in the circuitry – particularly because the electrodes were not restricted to any particular part of the STN associated with any of the loops.

Overall, there is some dispute in the literature regarding the frequency of obsessive and compulsive symptoms as a part of the Parkinson’s phenotype – and what their causes might be. Nevertheless, it seems that PD and its treatments, while both should be studied further, do provide promising avenues for exploring hemispheric bias in OCD as well as the role of dopaminergic pathways.

#### Evidence for obsessive and compulsive symptoms in Huntington’s disease

**Fig. 4:**
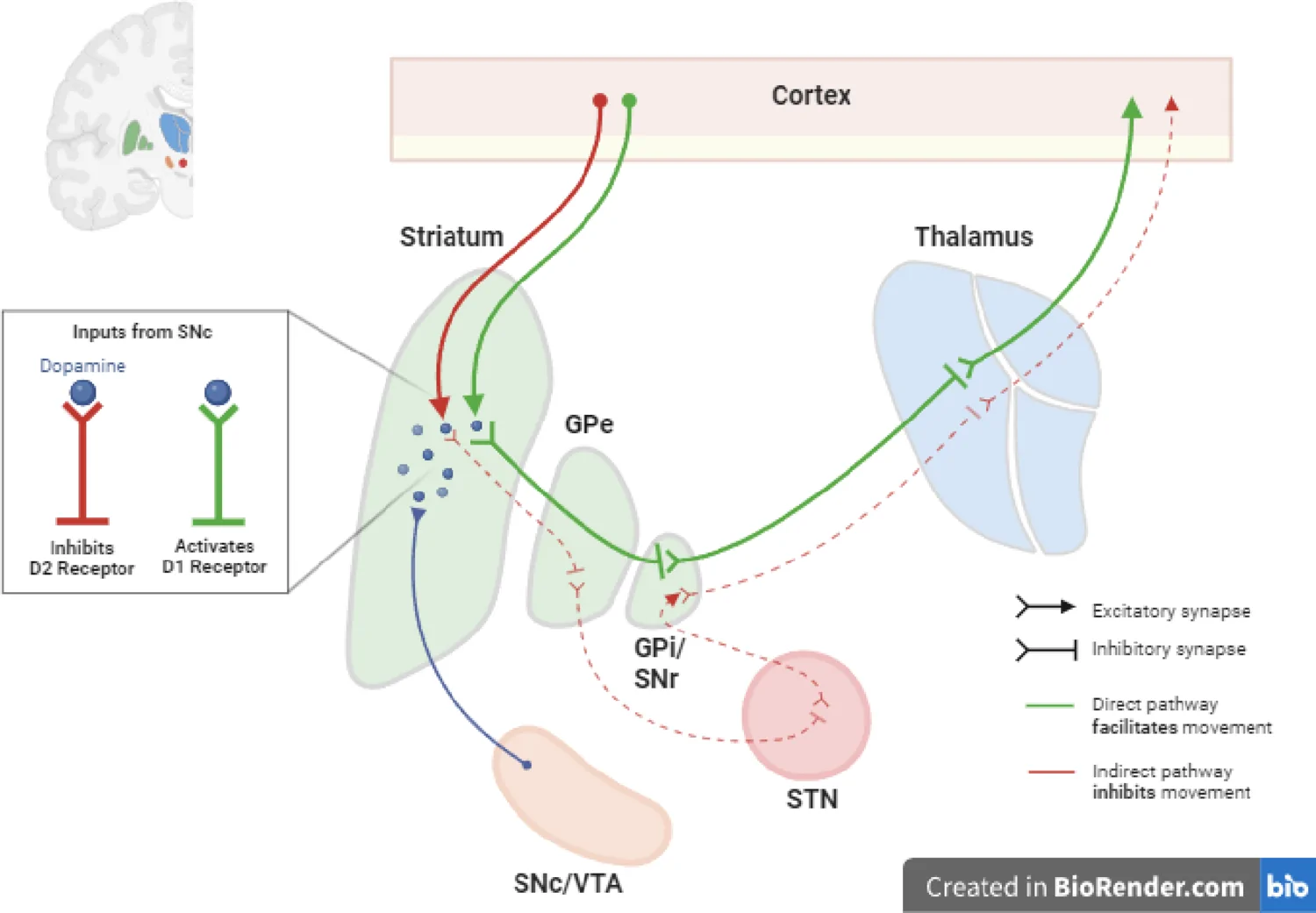
A schematic representing the pathological changes to the basal ganglia in Huntington’s disease. Dashed lines indicate degeneration of pathways. Adapted from ‘Direct and Indirect Pathways of the Basal Ganglia’, by BioRender.com (2024). Retrieved from https://app.biorender.com/biorender-templates. Adapted from https://app.biorender.com/biorender-templates

HD is a rare neurodegenerative disorder caused by autosomal dominant mutations in the huntingtin gene (Jimenez-Sanchez et al. 2017). Exactly how huntingtin mutations result in neurodegeneration is unknown, but suggestions include the formation of neuronal aggregates impairing the ubiquitin-proteasome pathway which is responsible for cellular homeostasis, as well as binding with other essential proteins e.g. CREB building protein to decrease their cellular availability (Schulte and Littleton 2011). The first symptoms, although they are subtle and therefore often go unnoticed, are largely psychiatric ones such as personality and behavioural changes (Rüb et al. 2016). Later clinical signs include more severe cognitive impairment and jerky movements or clumsiness, which are largely attributed to the preferential destruction of neurons projecting from the DS to the GPe (and therefore degeneration of the indirect ‘no go’ signal of the motor loop) (Fig. 4) (Walker 2007).

Obsessive-compulsive symptoms in HD are common. One study found that among 960 patients, 22.3% presented with such traits (Marder et al. 2000). Detailed case studies also exist delineating the psychiatric symptoms of HD patients, namely Cummings and Cunningham’s (1992) review of two patients with HD and OCD, the latter starting after the onset of the former.

Neuronal loss seems to start in the medial caudate region, later progressing to more lateral areas (Vonsattel et al. 1985). This is particularly notable as both the orbitofrontal and limbic loops project to the ventromedial caudate (as well as the NAc for the latter), and OCD may predate or coincide with more obvious motor and affective problems in HD (Molano-Eslava et al. 2008). All of this evidence lends itself to the hypothesis that obsessive-compulsive neuropsychiatric symptoms in HD can be attributed to orbitofrontal and ACC striatal circuit dysfunction – similar areas to those posited to be dysfunctional in OCD.

Anderson et al. (2010) later showed that OCS increase in severity in proportion to other clinical symptoms of HD, including motor and cognitive dysfunction. This is supported by data from Beglinger et al. (2007), who demonstrated that the probability of obsessive-compulsive symptoms is exacerbated as HD progresses. OCS measures are three times greater in patients with motor symptoms than at-risk patients with no motor symptoms. Again, linking this to patterns of degeneration, it is likely that this is due to exacerbation of caudate atrophy over time (Aylward et al. 2000).

Equally, it is clear that not every HD patient develops signs of OCS. Anderson et al. (2001) suggested in light of this that, while all HD patients have frontostriatal pathology, only those who have specific anterior and lateral OFC, and ventromedial caudate involvement, may go on to develop OCS. It is unclear however what might cause a biasing of dysfunction of those particular circuits in certain patients over others.

#### Evidence for obsessive and compulsive symptoms in PANDAS

PANDAS are characterised by rapid onset OCD and/or tic symptoms in childhood. Importantly, these symptoms have an episodic, waxing and waning course and most patients will fully recover, while others develop more persistent conditions – often after recurrence of the infection (Dop et al. 2021). PANDAS is generally considered to originate from autoimmunity against neural structures which develops after group A beta-haemolytic streptococcal infections (Moretti et al. 2008).

When discussing PANDAS, however, it is important to highlight the contentious nature of the diagnosis, as well as the significant revisions it has undergone over time. Its very existence and causes remain contentious (Harris and Singer 2006; La Bella et al. 2023), with numerous studies showing little to no relationship between tic and obsessive-compulsive symptom exacerbations and group A streptococcal infections (Kurlan et al. 2008; Leckman et al. 2011). Evolution in the diagnostic criteria compared to the NIMH group’s original working guidelines, and the distinct lack of reliable biological markers to confirm diagnosis (Orefici et al. 2016), have further muddied the waters when it comes to PANDAS. A clear example of this is the unclear boundary between PANDAS and other abrupt onset paediatric acute-onset neuropsychiatric syndromes (PANS) (Falcini et al. 2014). Confusions like these have resulted in inconsistent referrals, misdiagnosis, and at times overdiagnosis (Helm and Blackwood 2014), which will then inevitably complicate sampling by introducing misclassification biases in PANDAS research.

Despite these controversies, studies using ELISA and Western blotting have detected anti-basal ganglia antibodies (ABGA) in the serum of children with PANDAS but not controls, suggesting that, where the condition is present, these structures are particularly affected – although further study is needed to determine whether these antibodies truly are pathogenic or an epiphenomenon (Dale et al. 2005). Similarly, when comparing a group of children with PANDAS to a group with active but uncomplicated infections, Pavone et al. (2004) found a higher incidence of anti-brain antibodies in the former. This indicates that autoantibody presence cannot be accounted for only by streptococcal infection – indeed, there may also be a genetic component determining whether PANDAS develops secondary to infection or not. In line with this, Lougee et al. (2000) found higher rates of tic disorders and OCD in first-degree relatives of children with PANDAS compared to the general population, suggesting that streptococcal infection may precipitate a genetic predisposition to OCD in certain individuals.

Further adding to the autoantibody hypothesis of PANDAS, plasma exchange procedures and administration of intravenous immunoglobulin, which are thought to lead to clearance of cross-reactive antibodies (Kirvan et al. 2003), have both been demonstrated as effective in reducing neuropsychiatric symptoms in PANDAS (Perlmutter et al. 1999). To investigate which structures within the basal ganglia may be targeted, studies in mice have suggested that autoantibodies may be specific to the D2 receptor, implicating abnormal DA signalling in the aetiology of PANDAS (Cox et al. 2013). On the other hand, other studies have found no evidence for the presence of autoantibodies in PANDAS (see Harris and Singer 2006).

Neuroimaging studies are also consistent with the hypothesis that PANDAS arise as a result of an autoimmune reaction against the basal ganglia. Giedd et al. (2000) in their MRI study found that children with infection-associated OCD and tics had a greater average caudate, putamen, and globus pallidus size compared to healthy controls. Looking at differences in grey and white matter patterns, Cabrera et al. (2019) found alterations in PANDAS in the cortex, subcortex, and cerebellum – and most notably in the frontal lobes and basal ganglia. For the latter, as in the Geidd paper, they found increased grey matter volume, but interestingly reductions in white matter, possibly implicating issues with communication between the striatum and other areas of the brain in the emergence of OCS. It is important to note here that these findings do contrast with neuroimaging studies of idiopathic OCD, where striatal volume seems to be decreased. In PANDAS, the enlargement seen in neuroimaging studies may also be due to inflammation in the area (Kumar et al. 2015), which would similarly disrupt normal functioning. As for imaging of other brain areas, Zheng et al. (2020) found increased amygdala diffusivity, which, given its position in the limbic loop of the basal ganglia, indicates potential dysfunction in this circuit as a cause for OCS in PANDAS.

There have been a number of interventions tried to treat PANDAS. Plasma exchange and intravenous immunoglobulin have already been described, but other treatment options which are more similar to those for OCD include psychiatric care and neuropharmacology. One study examined the effects of CBT on a small group of children with PANDAS, the majority of whom were also taking SSRIs (Storch et al. 2006). A later review of the symptoms showed clear improvement in around half of the subjects. As for the effects of SSRIs alone, some reports suggest a positive effect on symptoms, while others draw very limited results – this is expected to be similar to cases of idiopathic OCD, where a significant proportion of treated patients see unsatisfactory improvement in symptoms (see Moretti et al. 2008).

#### Evidence for obsessive and compulsive symptoms in Sydenham’s chorea

SC is another disorder caused by basal ganglia-targeting autoimmunity after childhood infection with a group A beta-haemolytic streptococcus as a manifestation of rheumatic fever (Vasconcelos et al. 2019). Neurological symptoms are also abrupt and sudden in onset, typically involving chorea, after which the disorder is named. This involves non-rhythmic and often explosive, jerky involuntary movements, hypotonia, and more subtle signs such as grimacing and loss of fine motor control, all of which disappear in sleep. There are also distinctive psychological features apparent in many (but not all) patients, including anxiety, attention deficits, and obsessive-compulsive signs that may precede motor symptoms (Bonthius and Karacay 2003). In a comparison of OCS in patients with SC compared to those with rheumatic fever without SC, the former group presented with higher OCS scores (Swedo et al. 1989).

Given the similarities between SC and PANDAS, misdiagnoses can often be made between the two – however in general we can consider that differences lie in the types of movement demonstrated (choreatic in SC and choreiform in PANDAS (van Toorn et al. 2004), and the prevalence of obsessive-compulsive symptoms (necessary for diagnosis of PANDAS but not SC). The movement differences may be accounted for by different autoantibody affinities. In particular, antibodies in both PANDAS and SC seem to be targeted towards D2 and CaM kinase II – an enzyme involved in signal transduction pathways that can lead, amongst other things, to DA release (Kirvan et al. 2006). This group also found that there was less activation of CaM kinase II in response to sera from PANDAS patients compared to SC patients, therefore suggesting that perhaps more enzyme activity is required to trigger choreic movements, while smaller levels of activation contribute to choreiform movements and behavioural changes including OCD (da Rocha et al. 2008). Adding on to theories of DA pathway involvement in the psychiatric symptoms of SC, Nausieda et al. (1983) found evidence of DA hypersensitivity after development of SC, which may relate to both motor and behavioural signs through overactivation of the direct pathways of the motor and associative/limbic basal ganglia loops.

On a larger scale, the neurological changes seen in SC include clear striatal damage (Greenfield and Wolfsohn 1922) – which reflects the fact that Husby et al. (1976) found antibodies to the caudate and subthalamic nuclei in 46% of SC patients’ sera, the concentrations of which appeared to correlate with the severity of the chorea. More recent studies have also shown enlargements in the size of the putamen bilaterally (Karalok et al. 2021), aligning with the role of the putamen in the motor loop of the basal ganglia and the characteristic motor dysfunction in SC. As for activity levels, several studies indicate heightened perfusion of the basal ganglia, as well as hypermetabolism in the caudate and putamen (reviewed by Ehrlich and Walker 2017).

Overall, reviewing the data from both PANDAS and SC, we can see how the two might be considered as ends of a spectrum. OCS seem to be more prevalent in the former than the latter, while motor dysfunction is more prominent in SC. These differences may be reflected in the neuroimaging data and other evidence – as such, we might suggest that the obsessive-compulsive aspects of both disorders are related to greater caudate pathology (seen more in PANDAS than SC) while the motor symptoms are more attributable to putamen dysfunction (more common to SC than PANDAS). More evidence is required to confirm whether this is the case – it would be interesting in the future to conduct a study comparing the neuroanatomies and sera of patients with PANDAS, SC with OCD, and SC without OCD to see if there are any differences in the concentrations of autoantibody to particular parts of the basal ganglia.

#### Evidence for obsessive and compulsive symptoms in Tourette’s syndrome

TS is defined by vocal and motor tics varying in complexity that cause some level of distress or dysfunction (and can be voluntarily suppressed, although this is with discomfort) (Ueda et al. 2021). The tics are often preceded by ‘sensory tics/premonitory urges’, which are relieved by the performance of a motor tic (Martino et al. 2018). This could be paralleled to OCD, where the sensory tics correspond to obsessions and the motor tics to compulsions. In fact, obsessive-compulsive symptoms have for a long time been described in TS, with Gilles de la Tourette himself noticing behaviours and traits reminiscent of OCD in one of his original patients (Stevens 1964).

TS has significant comorbidity with OCD: it is estimated that OCD occurs in around 50% of TS patients (Frankel et al. 1986). A significant amount of the literature on the relationship between TS and OCD discusses heritability and genetic overlap. For example, Pauls and Leckman (1986) concluded through their family analysis study that OCD and TS are etiologically related. More recently, Davis et al. (2013) analysed the genetic correlations between TS and OCD, obtaining scores of 0.41 (whereby a score of 0 indicates complete independence in the genetic aetiology of a condition and 1 suggests that the genetic variants have identical influences in both cases). These results suggest some genetic overlap between the conditions.

Working theories for the mechanisms behind TS involve dysfunction of the CBGTC loops, leading to undesired thalamic activation and resultant involuntary movements and urges which may correspond to both the motor and psychiatric aspects of the syndrome (Morand-Beaulieu et al. 2017). Several neuroimaging papers support these theories, such as Braun et al. (1993), who in a PET study found evidence of reduced metabolism at rest, particularly in the orbitofrontal and paralimbic areas, and the basal ganglia. fMRI studies also offer interesting results, such as in the Neuner et al. (2014) study, where the group examined brain activity at various points prior to tic onset. Results two seconds before tic onset showed activation peaks in the supplementary motor area, primary motor cortex, and primary sensorimotor cortex, while one second before the tic starting there was activation of the ACC and putamen amongst others. With the onset of the tic itself, fMRI images showed activation of the thalamus, and primary motor and somatosensory cortices. This study, corroborated by results from Bohlhalter et al. (2006), follows in spectacular detail the motor cortico-striato-thalamo loop which has been hypothesised to be abnormal in TS. Adding to this, Peterson et al. (1999) showed that stimulation of the supplementary motor area causes similar ‘premonitory urges’ to those that are characteristic of TS – further implicating this structure in the leadup to a tic. The role of the insula must also be emphasised in these: in the Bohlhalter study, they found that just before tic onset a network of limbic areas including the insular cortex were activated. These findings align well with the role of the insula in interoceptive signalling, as well as suggesting a potential role for the limbic (alongside the motor) CBGTC loop in TS (which corresponds well with limbic loop abnormality in OCD as well, possibly underlying the correlation between these conditions) (Cavanna et al. 2017).

In an attempt to observe the striatum downstream of the cortical areas, Peterson et al. (2003) showed reductions in caudate and putamen volumes in the MRIs of TS patients relative to healthy controls. Maia et al. (2008) in their review propose, in light of this data, a development from the excessive DA hypothesis of TS: that tics in TS could be a result of an imbalance in the Go VS NoGo pathways of the motor loop in TS, the former dominating, resulting in the expression of partly involuntary movements as tics.

Supporting this idea, neuropharmacological studies have shown DA antagonists to be successful in the treatment of TS – particularly D2 blockers (Feinberg and Carroll 1979). These would increase activation of the indirect pathway, thus lending credence to the hypothesis that TS arises as a result of overactivation of the direct pathway relative to the indirect one. Furthermore, symptoms of TS are exacerbated on admission of drugs such as amphetamines, which increase DA transmission.

Maia and Frank (2011) also touch upon the effects of habit reversal training on TS prognoses, which has proven to be a successful treatment option for TS (Dutta and Cavanna 2013). This involves activation of tic-eliciting states while preventing tics from actually being performed. This would then lead to unlearning of the association between tic-eliciting states and the tics themselves. If this account is correct, it is distinctly similar to models of learning and unlearning discussed in theories of OCD that justify CBT as a form of treatment.

Based on the above evidence, it seems that one way of thinking about TS is as almost the opposite to PD – it seems to be a disorder largely driven by dopaminergic signalling dysfunction in the basal ganglia, in particular leading to an imbalance in the direct versus the indirect pathways (especially in the motor loop) that favours the former and thus creates a hyperkinetic disorder. PD is contrastingly caused by the degeneration of DA-producing neurons that results in an imbalance of activity that allows the indirect pathway to dominate instead, and thus generates a hypokinetic phenotype. OCD, meanwhile, could be subserved by a similar pathogenic mechanism to TS, but instead of the motor loop being predominantly affected, the orbitofrontal and limbic ones are.

## Discussion

### What does evidence from other basal ganglia disorders tell us about the role of the basal ganglia in OCD?

**Table 1:** A summary of the findings of studies reviewed in this article investigating neuroimaging and treatments for each of the basal ganglia disorders

OCD	Neuroimaging	Smaller left ACC and bilateral OFC (Rotge et al. 2009) Smaller caudate (Robinson et al. 1995) Increased metabolic activity of caudate and OFC (Baxter et al. 1987) Hyperactivity of the ACC (Maia et al. 2008) Decreased reward anticipation and activation of the ventral striatum in a monetary reward task (Figee et al. 2011) Symptom provocation leads to activation of the ACC and OFC loops (Rotge et al. 2008) Abnormal putamen morphology (Isobe et al. 2021) Heightened DA levels in the striatum compared to healthy controls (Denys et al. 2004)
	Treatments	Removal of the ACC, anterior limb of the internal capsule, or subcaudal areas (Balachander et al. 2019) Deep brain stimulation of the NAc (Denys et al. 2010) Deep brain stimulation of the anterior limb of the internal capsule, inferior thalamic peduncle, or STN (Kohl et al. 2014) DA modulators, which seem to decrease abnormal increases in activity in the ACC (Murray et al. 2019) SSRIs (Insel et al. 1985) Exposure and response therapy (Hezel and Simpson 2019)
Parkinson’s disease	Neuroimaging	SNc degeneration Possible right caudate hyperactivity (Mataix-Cols et al. 2004) – although this is inconclusive
	Treatments	Levodopa or DA agonists – administration of which often results in compulsive behaviours e.g. punding (Jankovic 2008)
Huntington’s disease	Neuroimaging	Progressive destruction of dorsal striatum, starting from medial caudate and progressing laterally (Vonsattel et al. 1985) Atrophy of the caudate (Aylward 2000)
PANDAS	Neuroimaging	Inflammation of caudate, putamen, and globus pallidus (Giedd et al. 2000) Increased grey matter but decreased white matter in the basal ganglia (Cabrera et al. 2019) Increased amygdala diffusivity (Zheng et al. 2020)
	Treatments	CBT and SSRIs (Storch et al. 2006) Plasma exchange and intravenous immunoglobulins (Kirvan et al. 2003)
Sydenham’s chorea	Neuroimaging	DA hypersensitivity in the basal ganglia (Nausieda et al. 1983) Striatal damage (Greenfield and Wolfsohn 1922) Bilateral increases in the volume of the putamen (Karalok et al. 2021) Increased basal ganglia perfusion and hypermetabolism of the caudate and putamen (Ehrlich and Walker 2017)
Tourette’s syndrome	Neuroimaging	Decreased metabolism at rest in the OFC and paralimbic areas (Braun et al. 1993) Increased activity of the SMA, primary motor cortex, and primary sensorimotor cortex 2s before a tic, increased activity of ACC and putamen 1s before a tic, and increased activity of the thalamus and primary motor and sensorimotor cortices at tic onset (Neuner et al. 2014) Decreased caudate and putamen volume (Peterson et al. 2003)
	Treatments	DA antagonists (Feinberg and Carroll 1979) Habit reversal training (Maia and Frank 2011)

Despite some inconsistencies in the literature, the evidence strongly points towards OCD being a disorder of the basal ganglia. A significant amount of this evidence comes from looking at idiopathic OCD – however, comparing the disorder to other conditions where OCS appear has also helped to confirm where problem areas may lie.

The data explored throughout this review and summarised in Table 1 supports the hypothesis that obsessions and compulsions may be subserved principally by dysfunction in the limbic and lateral OFC loops, and the goal-directed system of the DMS respectively. Considering compulsive behaviour first: compulsive symptoms were more prominent over obsessive ones in the disorders affecting primarily the dorsal striatum – notably HD, SC, TS, and treated PD. In HD, neuroimaging studies have suggested dysfunction in the caudate nucleus (Aylward et al. 2000), and therefore the DMS, paralleling its involvement in SC and TS (Ehrlich and Walker 2017; Peterson et al. 2003). Many of these movement disorders also show signs of increased putamen/DLS activity (Ehrlich and Walker 2017) and/or alterations in shape which mimic those seen in idiopathic OCD (Isobe et al. 2021). These findings therefore could support the hypothesis that compulsions in OCD may be mediated by a shift from goal-directed behaviours (mediated by the DMS/caudate) to habitual ones (the DLS/putamen).

In PD, treatment with DA agonists, which increase activity of the direct pathway relative to the indirect one, results in heightened motor activity and compulsive-like symptoms such as punding (Rajalingam and Fasano 2023). This is paralleled in HD where compulsivity arises secondary to degeneration of the indirect pathway. Even in idiopathic OCD evidence points towards heightened levels of DA in the basal ganglia (Denys et al. 2004), potentially implicating dominance of the direct over the indirect pathway in the motor loop in OCD secondary to increases in basal ganglia DA concentrations. This is further supported by evidence from treatments of TS using DA antagonists.

The evidence suggests that abnormalities in the limbic loop particularly subserve the aberrant emotional regulation and error or reward processing that drive obsessive thinking (Milad and Rauch 2012). This corresponds well with known functions of the ACC which feeds into the limbic loop, including error monitoring and sensitivity to threats (Bush et al. 2000). As for the role of the lateral OFC circuit, the fear and anxiety extending from abnormalities in the limbic loop could be supported by difficulties in flexible thinking and decision-making from the lateral OFC (Gaikwad 2014), as well as degeneration in the caudate, which it supplies. This theory is in line with the roles of the lateral OFC in flexible cognition, response inhibition, and escape from punishment (Milad and Rauch 2012). The proposed roles of the OFC and ACC in obsessions are supported, for example, by the fact that OCS often appear before motor symptoms in HD, aligning with observed patterns of degeneration starting in the medial caudate (Vonsattel et al. 1985).

Again, aberrant DA pathways may be involved here, with overactivity of the direct pathway and a hypoactive indirect pathway in the loops, such that unselected thoughts are sent to the anterior cingulate and orbitofrontal cortices rather than filtered away. As such, individuals may get stuck in a reverberative loop that leads to obsessive thinking, which may underlie why obsessive thoughts are so hard to dispel in OCD (Maia et al. 2008).

SC and PANDAS are very similar conditions separated by primary involvement of motor symptoms and compulsivity in one and OCS in the other. It is particularly interesting to consider that the amygdala, involved in the limbic loop, is implicated in PANDAS (Zheng et al. 2020) but, as far as we are aware, has not been highlighted as abnormal in SC. More research into the damage to parallel CBGTC loops in both of these conditions may therefore prove useful for detangling their roles in OCD. Additionally, the development of more temporally and spatially accurate neuroimaging technologies may enable us to follow the neurological pathways from the initiation of an obsession to the execution of a compulsion, giving us a more detailed picture of the mechanisms at play.

### Can we separate the neurological mechanisms behind individual obsessive and compulsive symptoms?

Obsessiveness and compulsivity, as traits, are different from OCD – in OCD, these may be linked and form a positive feedback loop, rather than existing separately. It may therefore be unhelpful to observe the traits singularly and attempt to find discrete brain regions responsible for each, especially given the highly interconnected nature of the basal ganglia (Haber et al. 2000).

If we consider OCD to be a problem with reward circuitry we can see how dysfunction in the limbic and lateral orbitofrontal loops (posited to play a role in obsessions) can result in abnormalities in how value is assigned to different goals or rewards, including the reduction of anxiety. This in itself could then support the shift from goal-oriented to habitual behaviour that has been argued to underlie compulsions, as the reward outcome might become poorly represented such that automatic behaviours dominate instead (Kim and Hikosaka 2015).

From a neuroanatomical perspective, while in any of the conditions described above either obsessions or compulsions may dominate the symptoms, in all cases there is some level of overlap and both may be present. This overlap may be driven by the ascending nigrostriatonigral circuitry linking the limbic loops to the motor ones (Haber et al. 2000), or other connections such as those directly between the ACC and OFC and motor cortices (Fatahi et al. 2020; Haber et al. 2022).

Therefore, while it can be helpful to simplify the system for our models and understanding, it is important to acknowledge the deep complexity of neural networks in the basal ganglia, which ensure that, while some brain areas may be more associated with some functions than others, there is some overlap in the roles. Furthermore, different areas may also be associated with different forms of obsession and compulsion, which may in the future be categorised further. For now, however, the areas underlying obsessions and compulsions in OCD cannot be fully separated.

### What implications may current understanding of pathophysiology have for future OCD treatments?

The current first-line treatments for OCD are psychotherapy and psychoactive serotonin-modulating drugs – however, the way they work in OCD remains unknown. They are also nonspecific, with targets all over the brain rather than particular areas, resulting in significant side effects. Similarly, there is little research into how ERP or other forms of psychotherapy might affect the neural circuitry in OCD, and efficacy varies between patients.

Comparing aetiologies of different disorders, we may be able to translate certain treatments for one condition to others. For example, if DA-based hypotheses for OCD are correct, clinicians may have more grounding for the use of DA receptor antagonists in OCD to disinhibit the indirect pathway and inhibit the direct pathway of various implicated CBGTC loops, as is done in the pharmacological treatment of TS (Feinberg and Carroll 1979). This would require further testing of treatments across conditions to determine which are successful, when, and why.

Overall, understanding the underlying anatomical and functional changes underlying OCD may allow us to develop more targeted, refined drugs and treatments for OCD that, alongside being more consistent across patients, enable us to target the root cause of the condition rather than just the symptoms.

### Limitations of the studies referenced in this paper

Despite some strongly suggestive evidence across different studies for particular brain regions playing a part in OCD, there are some limitations to the studies described above which provide for areas of disagreement in the literature. Difficulty arises from the small group sizes and low statistical significance in many of these studies, as well as inconsistencies in scales used for measuring obsessive and compulsive symptoms, making comparisons between papers difficult. On top of this, many of the scales that are used (such as the MOCI and LOI) do not have any true diagnostic value and instead serve as descriptive self-estimation scales, bringing in some doubt as to whether the results of these studies can be compared to clinically diagnosed OCD – particularly in those that describe OCS rather than OCD itself.

Neuroimaging studies are similarly not without their limitations, with concerns about whether structural abnormalities reflect epiphenomena or actual causes of dysfunction. Furthermore, many of these are carried out using a region-of-interest approach, which can create problems as self-fulfilling findings accumulate in the literature and skew perceptions of OCD towards a basal ganglia hypothesis, when other regions which are simply less considered may also be implicated (Menzies et al. 2008).

Some symptoms denoted as ‘obsessional’ or ‘compulsive’ may instead be caused by other issues e.g. motor abnormalities such as slowness that are characteristic of PD may be causing what seems like compulsive and obsessional behaviour rather than basal ganglia abnormalities similar to those in OCD. This raises the question of whether the symptoms are comparable between OCD and other conditions, or whether it is a semantic issue whereby the words ‘obsession’ and ‘compulsion’ have become umbrella terms encompassing far more symptoms than what they are defined as in the context of OCD.

This is also particularly true in TS – some authors have suggested that what some describe as compulsions could instead be complex motor tics (Palumbo and Kurlan 2007), and that the ‘premonitory urges’ that are compared to obsessions may not in reality be very comparable, as suppression of tics in TS is associated with feelings of discomfort and tension while suppression of compulsions in OCD leads to feelings that can be labelled as anxiety (Müller et al. 1997). So, while it is certainly still probable that OCD and TS are linked disorders, the literature, through possible overdiagnosis of OCD in TS, may be misrepresenting the strength of the connection.

### Methodological limitations of this review

This review was completed in a narrative format, which, while allowing for more flexibility, readability, and nuanced analysis, also comes with some drawbacks. The main limitation of this methodology is its susceptibility to confirmation and publication biases. For instance, as is typical for narrative reviews in order to preserve flexibility and breadth, we did not use strict predefined inclusion or exclusion criteria. Furthermore, our search was restricted to English-language publications, which may have excluded relevant work published in other languages. We also acknowledge that, given their lack of a step-by-step method, narrative reviews are difficult to replicate, and as such another author’s review on the same topic may have a different outcome. Nevertheless, rather than offering definitive conclusions, the value of this review lies in identifying areas ripe for more targeted research, including future systematic reviews or meta-analyses.

## Conclusion

This review has examined the role of the basal ganglia as a potential neurological substrate for OCD and obsessive-compulsive symptoms. It has analysed a range of evidence, focussing on neuroimaging and clinical studies across five disorders which implicate the basal ganglia in their pathology. Despite some inconsistencies in the literature, it appears that the lateral orbitofrontal and limbic loops are particularly implicated in the development of the disorder, as well as a shift in the balance of the goal-directed dorsomedial striatal circuitry and habitual dorsolateral striatal circuitry, favouring the latter.

There are some gaps in the literature that warrant further research, including neuroimaging studies in SC and PANDAS particularly to identify structural differences in any areas feeding into the CBGTC loops, and how these differences may result in variations in the prevalence of obsessive compared to compulsive symptoms. More research is also needed into the effects of OCD treatments, especially ERP and pharmacological therapies, on the presence of OCS in parallel to any structural changes in the basal ganglia. This would help to determine not only whether OCD and OCS are comparable but also whether they are subserved by similar mechanisms and can be treated in the same ways.

It is important to highlight that basal ganglia disruption is neither necessary nor sufficient for the development of OCD. Areas outside of the cortico-striato-thalamo-cortical circuitry are also implicated in OCD, and indeed some idiopathic OCD patients show no abnormalities in the basal ganglia at all. The opposite is also true, with many individuals suffering from highly disruptive basal ganglia damage and no OCS. This indicates that other brain areas may also cause OCD, furthering the idea that many different mechanisms can lead to the same, or a very similar, disorder. The basal ganglia view of OCD described in part above could therefore be one of many underlying causes of the condition and understanding other structures or processes involved would significantly advance our understanding of the condition and ultimately lead to better treatments.

## Data Availability

This is a review article based on previously published literature. As such, data sharing is not applicable here as no new datasets were generated or analysed during the study. All cited data are available in the references provided.

## Abbreviations

ABGA: Anti–basal ganglia antibodies
ACC: Anterior cingulate cortex
CBGTC: Cortico–basal ganglia–thalamo–cortical
DA: Dopamine
DLS: Dorsolateral striatum
DMS: Dorsomedial striatum
DS: Dorsal striatum
GPe: Globus pallidus external compartment
GPi: Globus pallidus internal compartment
HD: Huntington’s disease
NAc: Nucleus accumbens
OCD: Obsessive–compulsive disorder
OCS: Obsessive and compulsive symptoms
OFC: Orbitofrontal cortex
PANDAS: Paediatric autoimmune neuropsychiatric disorders associated with streptococcal infections
PD: Parkinson’s disease
SC: Sydenham’s chorea
SNc: Substantia nigra pars compacta
SNr: Substantia Nigra pars reticulata
TS: Tourette syndrome
VS: Ventral striatum
VTA: Ventral tegmental area
